# In Vitro Megakaryocyte Differentiation and Proplatelet Formation in Ph-Negative Classical Myeloproliferative Neoplasms: Distinct Patterns in the Different Clinical Phenotypes

**DOI:** 10.1371/journal.pone.0021015

**Published:** 2011-06-15

**Authors:** Alessandra Balduini, Stefania Badalucco, Maria Teresa Pugliano, Denis Baev, Annalisa De Silvestri, Marco Cattaneo, Vittorio Rosti, Giovanni Barosi

**Affiliations:** 1 Department of Biochemistry, University of Pavia, Pavia, Italy; 2 Department of Biomedical Engineering, Tufts University, Medford, Massachusetts, United States of America; 3 Dipartimento di Medicina, Chirurgia e Odontoiatria, Università degli Studi di Milano, Unità di Medicina 3, Azienda Ospedaliera San Paolo, Milano, Italy; 4 ViroStatics, Sassari, Italy; 5 Biometric Unit, IRCCS Policlinico S. Matteo Foundation, Pavia, Italy; 6 Unit of Clinical Epidemiology and Centre for the Study of Myelofibrosis, IRCCS Policlinico S. Matteo Foundation, Pavia, Italy; Heart Center Munich, Germany

## Abstract

**Background:**

Ph-negative myeloproliferative neoplasms (MPNs) are clonal disorders that include primary myelofibrosis (PMF), polycythemia vera (PV) and essential thrombocythemia (ET). Although the pathogenesis of MPNs is still incompletely understood, an involvement of the megakaryocyte lineage is a distinctive feature.

**Methodology/Principal Findings:**

We analyzed the in vitro megakaryocyte differentiation and proplatelet formation in 30 PMF, 8 ET, 8 PV patients, and 17 healthy controls (CTRL). Megakaryocytes were differentiated from peripheral blood CD34^+^ or CD45^+^ cells in the presence of thrombopoietin. Megakaryocyte output was higher in MPN patients than in CTRL with no correlation with the *JAK2* V617F mutation. PMF-derived megakaryocytes displayed nuclei with a bulbous appearance, were smaller than ET- or PV-derived megakaryocytes and formed proplatelets that presented several structural alterations. In contrast, ET- and PV-derived megakaryocytes produced more proplatelets with a striking increase in bifurcations and tips compared to both control and PMF. Proplatelets formation was correlated with platelet counts in patient peripheral blood. Patients with pre-fibrotic PMF had a pattern of megakaryocyte proliferation and proplatelet formation that was similar to that of fibrotic PMF and different from that of ET.

**Conclusions/Significance:**

In conclusion, MPNs are associated with high megakaryocyte proliferative potential. Profound differences in megakaryocyte morphology and proplatelet formation distinguish PMF, both fibrotic and prefibrotic, from ET and PV.

## Introduction

Megakaryocytes and platelets, which are their progeny, are highly specialized cells that participate in hemostatic and inflammatory functions. Since each platelet lives only about 10 days, the platelet supply is continually renewed by production of new platelets from the maturation of megakaryocytes [1]. The most recognized model of platelet formation provides that it occurs in the bone marrow environment where megakaryocytes extend long filaments, called proplatelets, that protrude through the vascular endothelium into the sinusoid lumen, where the platelets are released [2]–[6]. Physiological evidence of proplatelet formation has been demonstrated by electron microscopy analysis [7] and, more recently, proplatelet formation and platelet release has been shown by multiphoton intravital microscopy in intact bone marrow from mice [8]. However, many aspects regarding the mechanisms underlying proplatelet extension and platelet release remain unsolved, especially in humans [9]. Consequently, insight into the pathogenesis of megakaryocyte related diseases as well as treatment options are missing. Among the diseases, myloproliferative neoplasms (MPNs), which include polycythemia vera (PV), essential thrombocythemia (ET) and primary myelofibrosis (PMF), represent one of the most severe clinical picture that is still incurable. In PV, megakaryocytes are increased in number and display characteristic morphological abnormalities, such as hyperlobated nuclei. They are distinguishable from those in ET, which typically tend to form loose clusters or to lie close the bone marrow trabeculae and often show a significant degree of pleomorphism with variable sizes. PMF is characterized by important hyperplasia and atypia of megakaryocytes, whose nuclei appear hypolobated and cloud-like [10],[11]. Available information on mutations of genes encoding tyrosine kinases and their pathways do not explain entirely the molecular pathogenesis of MPNs and this lack of information contributes to the slow development of effective treatments. This justifies the continuous search for new cellular and molecular aberrations that specifically characterize these disorders and could become targets of new therapies. Previous studies demonstrated that megakaryocyte hyperplasia in PMF is, most likely, the consequence of both the increased ability of CD34^+^ progenitors to generate megakaryocytes and the decreased rate of megakaryocyte apoptosis, as suggested by their over-expression of the antiapoptotic protein bcl-xl [12]. Moreover, aberrant proplatelet formation has been shown in bone marrow from patients with MPNs [13]. Overall, these data suggest that abnormal megakaryopoiesis is a key feature of MPNs in general and of PMF primarily. However, it is unknown whether the pathological mechanisms underlying MPNs are caused by intrinsic defects of megakaryocyte function or by abnormalities of the bone marrow microenvironment, which regulates megakaryocyte formation and function.

In this study we investigated the *in vitro* pattern of differentiation of megakaryocytes from circulating hematopoietic progenitors obtained from patients with different MPNs and the capacity of these *in vitro*-differentiated megakaryocytes to form and extend proplatelets.

## Results

### MPNs display increased numbers of differentiating megakaryocytes

**Figure 1:**
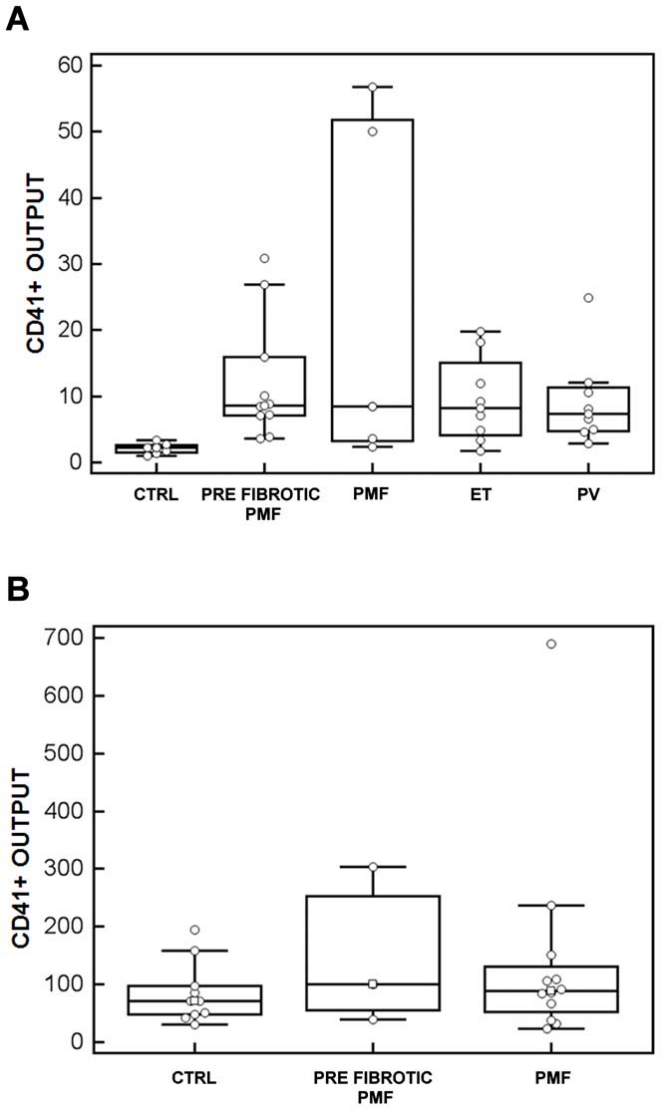
Box-and-whisker plots of megakaryocyte output in suspension cultures. CD45^+^ (A) and CD34^+^ (B) cells from peripheral blood were sorted as described in Methods and cultured for 14 days. At the end of the cultures, the yield of megakaryocyte was calculated as output of CD41^+^ cells with megakaryocyte morphology. CTRL: healthy controls; PMF: primary myeolofibrosis; ET: essential thrombocytemia; PV polycytemia vera. *p<0.01.

Megakaryocytes were derived from peripheral blood hematopoietic progenitor cells of 30 patients with PMF (13 pre-fibrotic and 17 fibrotic), 8 patients with ET, 8 patients with PV and 17 CTRL. CD45^+^ cell-initiated megakaryocyte cultures were performed in 13 patients with PMF (10 pre-fibrotic and 5 fibrotic), 8 patients with ET, 8 patients with PV and 7 CTRL (Figure 1A). The median output of CD41^+^ megakaryocytes at day 14 was 2.2% (range: 1.02–3.37) in CTRL, 8.61% in pre-fibrotic PMF (range: 3.6–30.86), 8.51% in fibrotic PMF (range: 2.33–56.71), 8.18% in ET (range: 1.7–19.8), 7.36% in PV (range: 2.8–24.9). Differences between MPN patients and CTRL were statistically significant (p<0.01), while differences among the 4 types of MPN were not statistically significant.

The *in vitro* production of megakaryocytes from CD34^+^ cells obtained from peripheral blood was studied in 15 patients with PMF (3 pre-fibrotic and 12 fibrotic) and 10 CTRL (Figure 1B). Immunomorphological analysis revealed that the median output of CD41^+^ cells with megakaryocyte morphology at day 14 was 71.14% (range: 30.1–193.9%) in CTRL, 99.75% (range: 39.8–303.8%) in pre-fibrotic PMF, 88.93% (range: 23.3–689.4%) in PMF. Although PMF progenitors displayed a trend towards increased capacity to generate megakaryocytes, the difference was not statistically significant among the three groups. This may be due to the higher variability in cell maturity and differentiation potential of mobilized CD34^+^ cells in CTRL [14]–[16].

Finally, no statistically significant differences in megakaryocyte output were observed between *JAK2* V617F and wild type *JAK2* patients (not shown).

### Megakaryocytes derived from pre-fibrotic and fibrotic PMF show peculiar characteristics compared to other MPNs and CTRL

**Figure 2:**
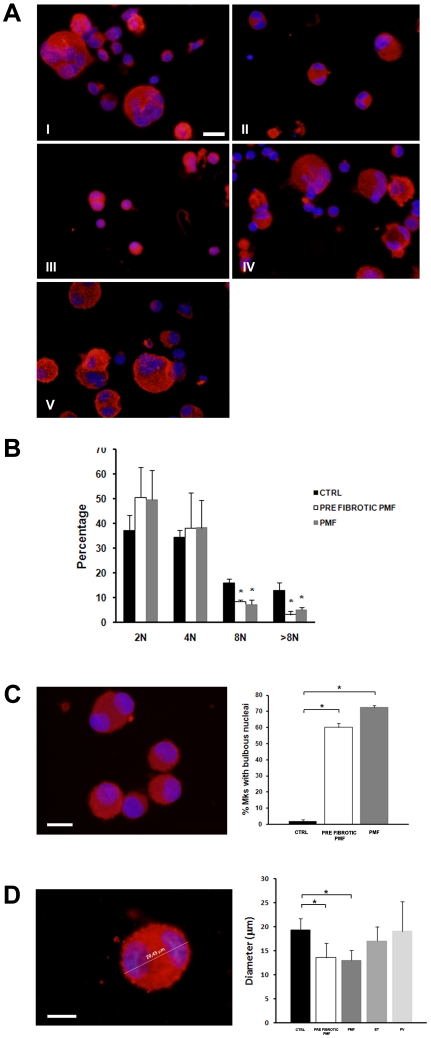
Characteristics of megakaryocyte morphology in MPNs and controls (CTRL). (A) Representative picture of differently shaped CD41^+^ (red) cells of controls (I), pre-fibrotic PMF(II), PMF (III), ET (IV) and PV (V). Nuclei are counterstained with Hoechst 33258 (blue). Scale bars are 15 µm. (B) Ploidy was analyzed as described in Methods. PMF derived megakaryocytes presented lower polyploidy with respect to controls. (C) PMF derived CD41^+^ megakaryocytes (red) displayed bulbous and hypo-segmented nuclei.(blue) (left panel). Scale bar is 15 µm. Means ± SD of the percentage of megakaryocytes displaying abnormal nuclei in pre-fibrotic PMF and PMF, as compared to control, are reported in the right panel. (D) Diameters of MPN derived CD41^+^ megakaryocytes (red) were analyzed as shown in the left panel and were performed as described in Methods. Scale bar is 10 µm. For each MPN category at least 100 megakaryocytes were analyzed, the means ± SD of diameters is reported in the right panel. *p<0.05.

Analysis of megakaryocyte morphology according to standard criteria [17] revealed significant differences in the maturation profile of PMF compared to PV, ET and CTRL, indicating a peculiar defect of megakaryocyte development in PMF compared to other MPNs (Figure 2A). Consistently, a lower percentage of PMF derived megakaryocytes was polyploid (>8N) compared to CTRL (Figure 2B). Moreover, the majority of PMF derived megakaryocytes presented bulbous nuclei, while almost no megakaryocytes from CTRL did (Figure 2C). Finally, diameter of megakaryocytes was measured at the end of the culture and prior to proplatelet formation. Megakaryocytes from PMF displayed a decreased diameter than those from ET, PV and CTRL (Figure 2D). Overall, our data confirm and extend previous observations [12], demonstrating that, although PMF generated more megakaryocytes, they were smaller and presented abnormal morphology of nuclei, compared to the other MPNs and CTRL.

In order to exclude that differences in megakaryocyte morphology were dependent on the maturation stage of progenitors derived from the different MPNs, a time course analysis was performed in PMF derived cultures. Progenitors derived from fibrotic and pre-fibrotic PMF were maintained in culture for 18 days and megakaryocyte morphology was analyzed. Results demonstrated that, even prolonging the culture incubation time, megakaryocytes derived from PMF remained smaller than CTRL and showed the same characteristics of immaturity observed when cultures were performed for shorter period of time (not shown). Overall these data demonstrate that impaired megakaryocyte development occurs in PMF, while in other MPN megakaryocytes mature normally.

### Proplatelet formation shows distinct abnormalities in the different MPNs and correlate with the clinical phenotypes

#### Studies in liquid culture suspension

**Figure 3:**
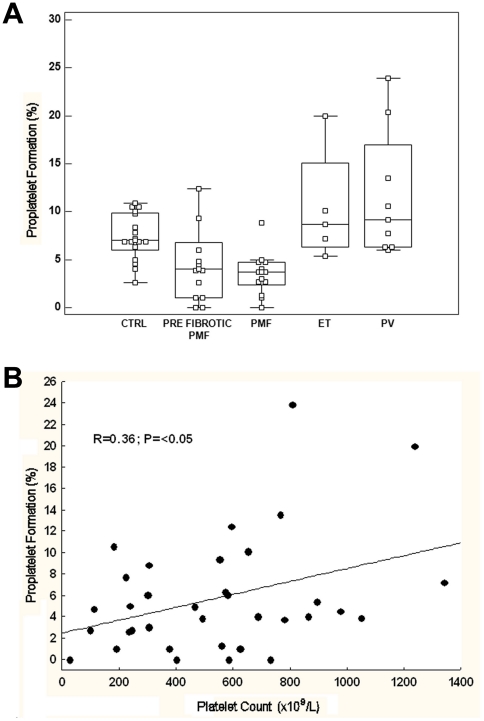
MPNs show important differences in megakaryocyte function and PPF. (A) Box-and-whisker plots of proplatelet output, expressed as percent of total megakaryocytes. (B) Correlation between the platelet count in peripheral blood and the number of proplatelets formed in culture in MPN patients. *p<0.01.

In order to explore whether defects in megakaryocyte development were associated to altered megakaryocyte function, we investigated the generation of proplatelets by MPN-derived and CTRL-derived megakaryocytes. Mature megakaryocytes, at the end of the culture, were reseeded and proplatelet formation was evaluated after 16 hours. In CTRL samples, a median of 7.5% (range: 2.6–11%) of megakaryocytes formed proplatelets, compared to 3.8% (range: 0–5%) of PMF-derived megakaryocytes, 8.65% (range: 5.5–20%) of ET-derived megakaryocytes and 9.15% (range: 6–23.9%) of PV-derived megakaryocytes (p = 0.001 for all the comparisons). No differences were observed between pre-fibrotic (median: 3.3%, range: 0–12.4%) and fibrotic PMF (median: 4.3%, range: 2.7–8.8%) (Figure 3A). There was a moderate correlation between the platelet count in peripheral blood and the *in vitro* proplatelet formation of MPN patients (r = 0.36; P = <0.05; Figure 3B). This was particularly evident in PMF: patients with thrombocytopenia (platelet count lower than 150×10^9^/L, N = 3) had the more severe defect in proplatelet formation (median 2.7%, range 0–4.7). The *JAK2* V617F mutation did not affect proplatelet formation neither in any category of MPN (not shown).

**Figure 4:**
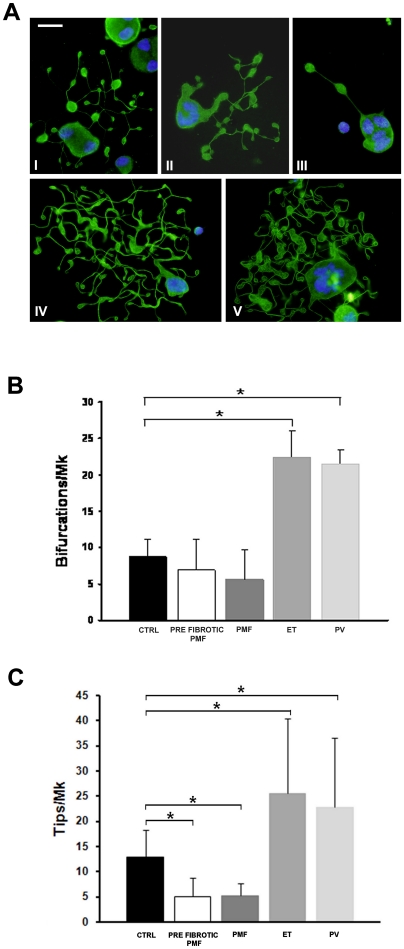
Analysis of proplatelet architecture. (A) Representative picture of differently organized proplatelet revealed by alpha tubulin staining (green) of controls (I), PMF (II–III), ET (IV) and PV (V). Nuclei are counterstained with Hoechst 33258 (blu). Scale bars are 15 µm. (B) Proplatelet bifurcations (means ± SD), which were identified upon immunostaining with an antibody against alpha tubulin. (C) Proplatelet tips (means ± SD), which were identified as coiled coil formations localized at the end of proplatelet branches. *p<0.05.

Nascent normal platelets form exclusively at the level of the terminal ends of the proplatelet shafts (i.e. the proplatelet tips) and the number of platelets that form is a function of the number of tips and shaft bifurcations [7]. We found that the proplatelets extended by PMF megakaryocytes presented several alterations with respect to CTRL. Specifically, proplatelets had a very variable numbers of bifurcations that frequently did not present any tips at the terminal end. Consequently, we observed a significant reduction of tips with respect to CTRL proplatelets, thus indicating a defect in proplatelet structure (Figure 4A–C). In contrast, ET- and PV-derived proplatelets displayed a striking increase in bifurcations and tips compared to both CTRL and PMF (Figure 4A–C). No other defects in proplatelet structure and tubulin distribution were observed (Figure 4A).

#### Studies in cell adhering to adhesive proteins

**Figure 5:**
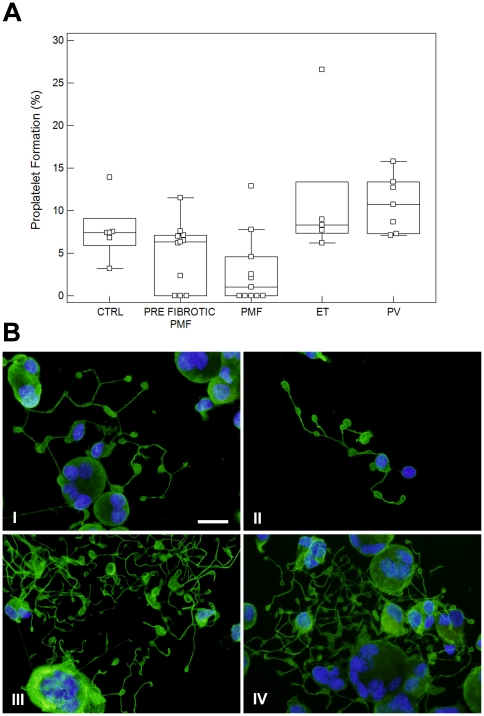
Proplatelet formation by megakaryocytes adhering to fibrinogen. (A) Box-and-whisker plots of proplatelet output, expressed as percent of total megakaryocytes, *p<0.01. (B) Representative picture of differently organized proplatelet revealed by alpha tubulin staining (green) of controls (I), PMF (II), ET (III) and PV (IV). Nuclei are counterstained with Hoechst 33258 (blu). Scale bars are 15 µm.

Experiments were also performed under conditions in which megakaryocytes were let to adhere to fibrinogen, an adhesive proteins that positively regulate proplatelet formation. In CTRL samples, a median of 7.4% (range: 3.2–13.9%) of megakaryocytes formed proplatelets, compared to 1% (range: 0–12.9%) of PMF-derived megakaryocytes, 8.3% (range: 6.2–26.5%) of ET-derived megakaryocytes and 10.7% (range: 7.1–15.8%) of PV-derived megakaryocytes (p = 0.01 for all the comparisons). No differences were observed between pre-fibrotic (median: 6.35%, range: 0–11.5%) and fibrotic PMF (Figure 5A). Further, as for suspension cultures, PMF-derived proplatelets showed a simpler structure as compared to CTRL, ET and PV (Figure 5B I–II). In contrast, proplatelets extended by ET- and PV-derived megakaryocytes displayed the same morphology observed in suspension cultures with an evident increase of shaft bifurcations and tips with respect to CTRL (Figure 5 III–IV). Taken together, these data demonstrate that the PMF-derived megakaryocytes present intrinsic defect in extending proplatelets that are independent from substrate regulation.

## Discussion

Megakaryocytes are large bone marrow cells that release platelets into the blood stream by elongating proplatelets [3]–[6]. Recent studies pointed to a key role of abnormal megakaryocytopoiesis in the pathogenesis of MPNs [12],[13], however , little is known about the latter stage of megakaryocyte development and proplatelet formation in these diseases. Therefore, we studied megakaryocyte differentiation and proplatelets formation *in vitro*, by culturing progenitor cells from in PMF, ET and PV patients, with the aim of establishing to what extent the observed abnormalities are attributable to intrinsic cellular defects [27]. Indeed, we found that each MPN category displayed peculiar alterations of megakaryocyte differentiation and function *in vitro*, suggesting that, besides the potential deregulation of bone marrow microenvironment, intrinsic defects of megakaryocyte function contribute to the pathogenesis of MPNs. Consistently with a previous report by Ciurea et al [12], we found that the *in vitro* megakaryocytopoiesis from progenitor cells derived from PMF, ET and PV patients was increased compared to healthy controls, with no statistically significant differences observed among the types of MPNs. Moreover, in MPNs the capacity of stem cells to generate more megakaryocytes was not associated with the presence of the V617F mutation of the *JAK2* gene [12]. These data suggest that other, yet-unknown, genetic mutations may contribute to altered megakaryopoiesis in MPNs [18]–[22]. Interestingly, PMF megakaryocytes were smaller than those of other MPN or of CTRL: these findings are in keeping with the well known morphological alterations of megakaryocytes that can be observed in bone marrow biopsies, which represent a key element for the diagnosis of the different types of MPN [23]–[26], [28]–[30]. Most importantly, our results could be correlated with recent data by Besancenot et al. that claimed that malignant megakaryocytes undergo abnormal proliferation by escaping the phisiological mechanisms of cell cycle arrest and senescence induced by TPO signalling [27].

PMF-derived megakaryocytes showed a defect in proplatelet formation, at variance with ET- and PV-derived megakaryocytes, which produced more proplatelets compared to CTRL-derived megakaryocytes. Moreover, a positive correlation between *in vitro* proplatelet formation and the platelet count in MPN patients' peripheral blood was observed. Moreover, consistently with the demonstrations that nascent platelets are formed exclusively at the level of the terminal ends of the proplatelet shafts (i.e. the proplatelet tips) and that the number of platelets that are produced is a function of the number of tips and shaft bifurcations [6], we documented that the number of bifurcations was very variable and tips for each proplatelet-bearing megakaryocyte were significantly decreased in PMF patients with respect to other MPNs and controls, while it was significantly increased in ET and PV patients. Of particular interest, we found that megakaryocytes from both pre-fibrotic and fully fibrotic PMF produce less proplatelets than normal. This finding underscores the biological diversity of prefibrotic PMF and ET, and supports the distinction between these two nosological entities, which has been proposed on the basis of bone marrow morphology [28]–[30] but has not been universally accepted yet [26].

We also studied proplatelet formation from megakaryocytes that were adhering to fibrinogen, an adhesive protein known to support proplatelet formation [31],[32]. Consistently with suspension cultures, a reduction in proplatelet formation was found in PMF-derived megakaryocytes, whereas an increase was observed in PV- and ET-derived megakaryocytes. Further, abnormalities in proplatelets architecture, observed in suspension culture-derived proplatelets, were also evident in adhesion to fibrinogen. These data highlighted the presence of intrinsic defect in megakaryocyte development that resulted to be independent from the culture environment and characteristic of each MPN category [33]–[38]. Interestingly, aberrant proplatelet formation was shown in histological sections of bone marrow from ET and PMF patients [13]. This observation represents a step forward our understanding of MPN bone marrow composition and suggests that altered regulation of proplatelet formation occurs in MPN bone marrow. Furthermore, our data extend these results demonstrating that MPN derived megakaryocytes present intrinsic defects in extending proplatelets that are abnormal both in numbers and structure. Therefore both set of date point to an aberrant regulation of proplatelet formation in MPN patients. Finally, our results are also strengthened by the direct correlation that we found between platelet count and number of proplatelets.

In conclusion, the results of our study provide important new elements in the understanding of the biology of megakaryocyte and proplatelet formation in MPN, and open a new perspective into the understanding of the pathophysiology of platelet production in these disorders. It represents the first step towards the understanding of basic cell biology and regulatory mechanisms of platelet formation in MPNs. Our results suggest that our experimental model may be useful for dissecting the pathogenesis of MPN, for identifying lesions responsible for disease evolution and for testing therapeutic agents [33]–[39]. The long-term goal is to utilize the model to elucidate new clinical options for disease management.

## Materials and Methods

### Ethics Statement

The policies for collection and use of blood samples were approved by the Institutional Review Board of the IRCCS Policlinico S. Matteo Foundation, and all patients gave consent for the donation of blood samples.

### Patients and controls

We studied 46 patients with MPNs (Table 1): 30 with PMF, 8 with ET and 8 with PV. All patients with PMF referred to the Center for the Study of Myelofibrosis of the IRCCS Policlinico S. Matteo Foundation in Pavia, Italy, between March 2007 and December 2009; none of them was receiving any disease-modifying therapy at the time of their enrollment in the study; however, patients with PV were all under treatment with phlebotomy in order to try to maintain their hematocrit below 45% (males) or 42% (females). In PMF, 17 of these patients met the 2008 WHO criteria for PMF, fibrotic type (fibrotic PMF) [28], while the remaining patients met the 2001 WHO criteria for pre-fibrotic PMF (granulopoiesis hyperplasia with predominance of immature and segmented forms, and high number and clustering of atypical megakaryocytes) [29] and had no or minimal grade reticulin fibrosis (EUMNET grading lower than 1) [30]. ET and PV patients were referred either to the Center for the Study of Myelofibrosis of the IRCCS Policlinico S. Matteo Foundation in Pavia or to the Dipartimento di Medicina, Chirurgia e Odontoiatria, Università degli Studi di Milano, Azienda Ospedaliera San Paolo in Milano. Diagnoses were based on the WHO criteria [28],[29]. A normal, age and sex matched, control population consisted in 10 healthy volunteers who were subsequently treated with granulocyte-colony stimulating factor (G-CSF) and 7 students or staff members, who had not been treated with G-CSF (see Table 1 for clinical and epidemiological data of healthy controls).

**Table 1:** Epidemiological and clinical data of patient and control populations*.

	PMF (n = 30)	ET (n = 8)	PV (n = 8)	CTRLs (n = 17)
Age	45 (24–56)	49 (24–56)	35 (33–38)	34 (21–54)
Sex (M/F)	19/11	5/3	7/1	8/9
Hb (g/dl)	12.3 (11.3–14.2)	14.6 (11.6–15.3)	17.6 (15.1–18.1)	13.9 (12.4–16.8)
Hct (%)	41.2 (39.1–45.7)	43.6 (39.9–49.8	47.2 (42.9–50.8)	44.3 (41.9–45.5)
WBC (×10^9^/L)	7.5 (1.9–12.8)	8.8 (5.3–12.8)	8.2 (6.7–13.7)	6.4 (4.9–8.2)
Plt (×10^9^/L)	524 (234–977)	780 (651–1340)	352 (181–807)	289 (199–341)
*JAK2* wt (n)	10	4	1	17
*JAK2* V617F (n)	20	4	7	0

*Age, Hb, Hct, WBC, and Plt counts are expressed as median (range). Diagnosis was made according to WHO criteria (see text for details). Values reported in the table are those at the moment in which blood was drawn for experiments. PMF and ET patients were at diagnosis or before the beginning of any cytoreductive therapy. Patients with PV were all under treatment with phlebotomy (but not with cytostatic therapy) in order to try to lower their Hct below 45% (males) or 42% (females). Healthy CTRLs hematological values were assessed before treatment with G-CSF (see text for details).

### Clinical and Laboratory Assessment

At the time of blood withdrawal for this study, the medical histories of the patients were collected. In all patients, blood samples were obtained to determine complete blood count and to examine peripheral blood smear for differential white blood cell count. Circulating CD34^+^ hematopoietic progenitor cells were counted using a standard methodology [40] The presence of V617F mutation of *JAK2* was determined using the allele specific-PCR assay on DNA purified from granulocytes, as reported [41]: samples were considered homozygous when the percentage of the mutant allele was greater than 50%.

### Differentiation of megakaryocytes and megakaryocyte morphological analysis

CD34^+^ or CD45^+^ cells from patients' and controls' peripheral blood samples were separated by immunomagnetic bead selection as previously described [31],[42]. CD45^+^ cells were separated from patients that presented low numbers of peripheral CD34^+^ cells (<10/µl) [42]. CD45^+^ and CD34^+^ cells were then cultured in Stem Spam medium (Stem Cell Technologies, Vancouver, Canada) supplemented with 10 ng/ml TPO, IL-6, and IL-11 (PeproTech EC Ltd, London, UK), at 37°C in a 5% CO_2_ fully-humidified incubator, for 14 days, as previously described [31],[42]. At day 14, 150×10^3^ cells were collected, cytospun on glass coverslips and stained with a primary antibody against CD41 (goat polyclonal anti-CD41, 1∶100, Santa Cruz Biotechnology, Heidelberg, Germany) to evaluate megakaryocyte output and maturation. After washing with PBS, cells were incubated with 10 µg/ml of an anti-goat Ig secondary antibody conjugated with Alexa Fluor 488 (Invitrogen, Milan, Italy) in PBS at room temperature (RT) for 1 hour. Nuclear counterstaining was performed with Hoechst 33258 (100 ng/ml in PBS) at RT for 3 minutes. Specimens were mounted in Pro Long Antifade Reagent (Invitrogen, Milan, Italy). Negative controls were routinely performed by omitting the primary antibody. Megakaryocytes were identified on the basis of CD41 expression, and assigned to distinct stages of maturation according to standard morphological criteria [17]. Megakaryocyte output was calculated as the percentage of CD41^+^ cells at day 14, and normalized to the total number of CD45^+^ or CD34^+^ cells obtained from peripheral blood at the beginning of the cell culture. Measurements of megakaryocyte diameters were performed on acquired images by the Axiovision 4.5 software (Carl Zeiss). At least one hundred megakaryocytes were analyzed for each sample [42].

### Proplatelet formation

Megakaryocyte yield and proplatelets were evaluated as previously described at the end of the cell culture [31],[42] both in culture medium and after adhesion of megakaryocytes to adhesive proteins. For studies in culture medium, large, mature megakaryocytes were separated from cultured cells at day 14 by sedimentation on a bovine serum albumin (BSA, Sigma, Milan, Italy) gradient (3–4%). For each subject, an aliquot of 1×10^5^ cells was replated and incubated for additional 16 hours. The percentage of megakaryocytes extending proplatelets at 16 h was assessed by phase contrast and immunofluorescence microscopy, using the Olympus BX51 fluorescence microscopy (Olympus Deutschland GmbH, Hamburg, Germany) and a 63×/1.25 UplanF1 oil-immersion objective. Proplatelet-bearing megakaryocytes were then cytospun on glass coverslips and double-stained with antibodies against CD41 and α-tubulin (clone DM1A, Sigma, Milan, Italy). Megakaryocytes forming proplatelets were identified as large CD41^+^ cells extending α-tubulin-positive long filamentous structures. The percentage of CD41^+^ cells bearing proplatelets was calculated. Evaluation of proplatelets by phase contrast and immunofluorescent microscopy resulted in superimposable results. For each specimen, at least 100 megakaryocytes were evaluated. The number of branching and platelet-like structures on each proplatelet-bearing megakaryocyte was calculated.

To analyze the formation of proplatelets from megakaryocytes adhering to adhesive substrates, 12 mm glass coverslips were coated with 100 µg/ml fibrinogen (FBG) (Sigma, Milan, Italy), for 2 hours at RT and subsequently blocked with 1% BSA for 1 hour at RT. Cells at day 14 of culture were harvested, plated onto substrate-coated coverslips in 24-wells plates (1×10^5^ cells/well), and allowed to adhere for 16 hours at 37°C and 5% CO_2_. Proplatelet formation was evaluated at 16 hours by phase-contrast microscopy and by fluorescence microscopy, as described above.

### Ploidy analysis

At the end of the cell culture, 5×10^5^ cells derived from PMF and CTRL peripheral blood were harvested and stained with a FITC-conjugated antibody against human CD41 (clone HIP8, BioLegend, California, USA) for 30 minutes on ice at dark. Then, cells were fixed in PFA 4% for 20 minutes at RT, permeabilized with 0,1% Tween 20 (Sigma, Milan, Italy) supplemented with 100 µg/ml RNAse (Sigma, Milan, Italy) and stained with 50 µg/ml Propidium Iodide (PI) (Sigma, Milan, Italy) for 30 minutes on ice at dark. Ploidy of megakaryocytes was evaluated by flow cytometry using a BD LSR II flow cytometer (BD Biosciences, San Jose', CA, USA) with DiVa 6.1 data acquisition software (BD Biosciences, San Jose', CA, USA). A minimum of 20000 events were collected in the CD41^+^ gate. Non-stained samples, FITC-isotype controls and fluorochrome minus one (FMO) controls were used to set the correct analytical gating. Off-line data analysis was performed using FCS Express 3.0 (DeNovo Software, Los Angeles, CA, USA) and ModFit LT (Verity, Topsham, ME, USA) software packages.

### Statistics

Values are expressed as mean ± SD or median (range), when appropriate. Analysis by Kruskall-Wallis test was followed by post-hoc testing using the critical difference of the mean ranks after Conover (Conover WJ, 1999, Practical nonparametric statistics, 3^rd^ edition, New York, John Wiley & Sons). A value of p<0.05 was considered statistically significant. Statistical analysis was carried out using SigmaStat 3.0 and Medcalc version 11.5 software. All experiments were independently replicated at least three times, unless differently specified.
